# The NMR structure of the Orf63 lytic developmental protein from lambda bacteriophage

**DOI:** 10.1038/s41598-024-54508-9

**Published:** 2024-02-15

**Authors:** Naushaba Khan, Tavawn Graham, Katarzyna Franciszkiewicz, Sylwia Bloch, Bożena Nejman-Faleńczyk, Alicja Wegrzyn, Logan W. Donaldson

**Affiliations:** 1 Department of Biology, York University, Toronto, ON M3J1P3 Canada; 2 Department of Molecular Biology, University of Gdańsk, 80-308 Gdańsk, Poland; 3 Phage Therapy Center, University Center for Applied and Interdisciplinary Research, University of Gdańsk, 80-822 Gdańsk, Poland

**Keywords:** Viral proteins, Solution-state NMR

## Abstract

The *orf63* gene resides in a region of the lambda bacteriophage genome between the *exo* and *xis* genes and is among the earliest genes transcribed during infection. In lambda phage and Shiga toxin (Stx) producing phages found in enterohemorrhagic *Escherichia coli* (EHEC) associated with food poisoning, Orf63 expression reduces the host survival and hastens the period between infection and lysis thereby giving it pro-lytic qualities. The NMR structure of dimeric Orf63 reveals a fold consisting of two helices and one strand that all make extensive intermolecular contacts. Structure-based data mining failed to identify any Orf63 homolog beyond the family of temperate bacteriophages. A machine learning approach was used to design an amphipathic helical ligand that bound a hydrophobic cleft on Orf63 with micromolar affinity. This approach may open a new path towards designing therapeutics that antagonize the contributions of Stx phages in EHEC outbreaks.

## Introduction

*Escherichia coli* O157:H7 has been associated with food- and water-borne infections throughout the world. While only a few percent of affected people are hospitalized, the impact can be large when infection leads to a community outbreak. Since the discovery of *E. coli* O157:H7 over forty years ago^1^, other clinically important strains have emerged^2^. One way in which pathogenic *E. coli* are genetically distinct from their benign counterparts is that their genome contains prophage DNA which bears *stx* genes, encoding Shiga toxins. Such a phage is called Shiga toxin-converting phage, or Stx phage, and as a prophage it remains dormant until a stress pathway is activated in the host bacteria leading to a developmental transition from a lysogenic state to an activated lytic state. As the phage replicates, it also produces one of two forms of the Shiga toxin enzyme (Stx1 or Stx2) that enters intestinal epithelial cells and kills them due to ribosome inactivation^3^. While the oxidative attack by intestinal immune cells offers one way to induce a stress response in bacteria, antibiotic treatment may also lead to the same outcome. As a result, antibiotics that would normally be the first treatment against a bacterial infection are avoided during a Shiga-toxin producing *E. coli* (STEC) infection. This critical limitation impresses a need to explore phage-bacteria dynamics further and identify new therapeutic stratagies^4,5^.

**Figure 1:**
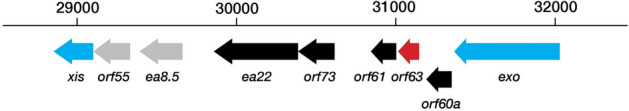
The *exo-xis* region of phage λ. This region may be considered in terms of a conserved and largely invariant region consisting of *orf60a*, *orf63* (red), *orf61*, *orf73*, and *ea22* followed by a region with a variable gene composition (grey).

Stx phages share many characteristics with phage λ, a model system associated with many landmark discoveries in molecular biology. One shared genetic region found between the *exo* and *xis* genes encodes five proteins (Orf60a, Orf61, Orf63, Orf73, and Ea22) that may work separately or together at the earliest stages of infection to affect the commitment to a lysogenic or lytic developmental outcome^6–8^ (Fig. 1). Originally, *exo-xis* genes were associated with cell cycle and DNA replication changes driven by the *p*_L_ promoter^9^ during the earliest stages of infection with phage λ^10^. Assays that measure the period between induction and the observation of new viral progeny, the number of bacterial survivors after infection, and the efficiently in which bacteria are converted to lysogens all suggest that Orf60a, Orf61, and Orf63 are pro-lytic where Ea22 and Orf73 tend to be pro-lysogenic^8,11^. Orf63, the subject of this report, is a 63 amino acid protein that is highly conserved among λ and Stx phages alike^12^ Since the effects of Orf63 and other *exo-xis* region gene products were amplified in STEC bacteria relative to benign *E. coli* strains infected with λ^13^, a closer examination may reveal new protein partnerships and pathways that Stx phages use to favor their success within their host.

Here, we present the structure of Orf63 as a first step towards understanding its functional role. This investigation increases the repertoire of *exo-xis* proteins that have either been modeled or solved to date^14,15^. Through molecular modeling, we have identified a region on Orf63 that can bind a short α-helical ligand. Protein partners of Orf63 may share a similar binding mode. Orf63 dimerizes in a way that has not been previously observed in the Protein Data Bank. A search of predicted protein structures from comprehensive databases suggests the Orf63 dimeric fold is exclusive to phage proteins.

## Results

Orf63 is a small protein that is known as DUF1382 in the Conserved Domain Database (CDD) and as IPR009814 in the InterPro protein family database as IPR009814. Size exclusion chromatography multi-angle laser scattering (SEC-MALS) revealed that Orf63 occurs exclusively as a dimer in solution (Supplementary Fig. S1). The Orf63 protein was the most soluble and produced the highest quality NMR spectra at pH 7.5. Following this preliminary survey, the NMR structure of 6xHis-Flag affinity tagged Orf63 was determined using a conventional combination of experiments supplemented with a ^13^C-filtered, ^13^C-separated NOESY experiment to identify intermolecular contacts. The PDB entry 8DSB includes a 6xHis-Flag affinity tag adding 14 residues to the coordinate numbering (Supplementary Fig. S2). Representative NOESY spectra from the methyl groups of Val27, Ile29, and Leu44 making key intra- and intermolecular contacts within the hydrophobic core are provided in Supplementary Figs. S3–S5. A total of 796 distance restraints, 25 hydrogen bond restraint pairs, and 30 torsion angle restraints were used as input for a two-stage structure calculation that used CYANA to produce an initial ensemble and Rosetta for final refinement. A complete statistical summary of the structure determination is provided in Supplementary Table S1. A flowchart of the experimental strategy is presented with more detail in Supplementary Fig. S6 and scripts used to perform Rosetta based refinement are presented in Supplementary Figs. S7 and S8. Overall, the RMSD for ordered regions of the ensemble of the 20 lowest energy structures was 0.8 Å for backbone atoms and 1.1 Å for all heavy atoms.

**Figure 2:**
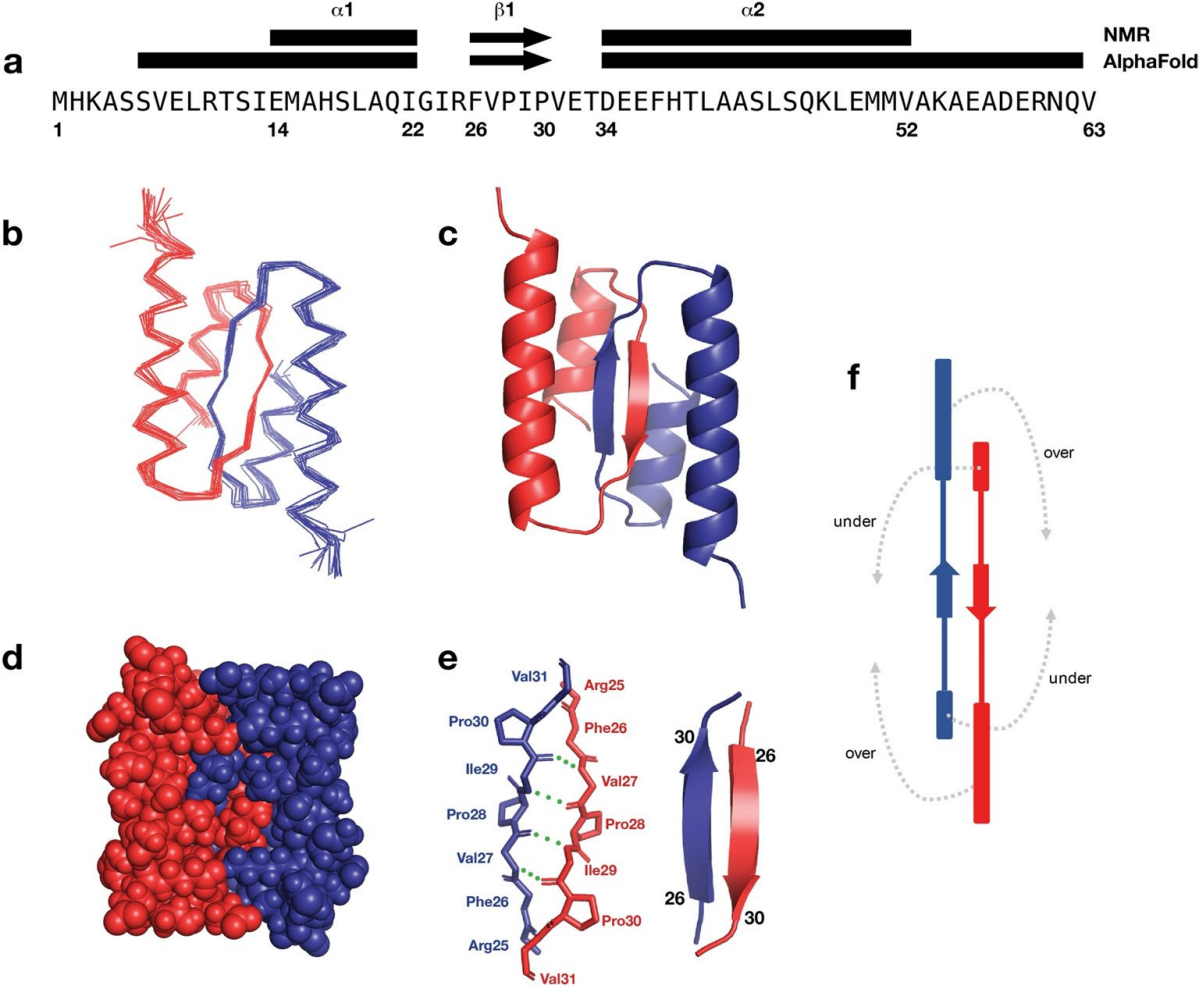
Structural characteristics of λ Orf63. (**a**) Secondary structural elements mapped onto the sequence from the NMR structure and AlphaFold model. (**b**) A Cα trace of the best 20 structures in the ensemble (**c, d**) A cartoon and space filling representation of the dimer (**e**) Hydrogen bond network across the β-strands at the dimer interface. (**f**) Topology diagram of the dimer. The fold may be considered in terms of the two α-helices folding under and over the β-sheet. Residue numbering follows the native sequence. To find the corresponding residue in the PDB coordinate file 8DSX, add 14 to account for non-native sequences arising from affinity tags (E36 = E50 in the coordinate file).

The structural features of λ Orf63 are summarized in Fig. 2. From the initial assignments, 11 amino acids from the N-terminus and 9 amino acids from the C-terminus were determined to be unstructured leaving a very compact dimeric fold consisting of two α-helices and one β-strand. The tightly intertwined dimer can be folded by placing two extended chains beside each other to form the anti-parallel, two-stranded β-sheet and then folding the two α-helices above and below the β-sheet. The dimer interface draws contributions from much of the protein making it unlikely that the protein would ever be observed in a monomeric state. Indeed, of the 43 residues that comprise the folded Orf63 protomer with a total accessible surface area of 4109 Å^2^, 27 residues participate in the binding interface with a total buried surface area of 1215 Å^2^. Detailed output from surface area analysis performed by PISA^16^ is provided as Supplementary Table S2. Within the central two-stranded β-sheet, two prolines are positioned in a manner that maintains a regular hydrogen bonding network supported a combination of backbone HN-HN, HN-HA, and HA-HN NOE observations. A survey of the PDB using SSM^17^ and FoldSeek^18^ revealed no homologous fold. Extending the survey to large databases whose structures have been predicted by AlphaFold, the only homologous examples were other viral Orf63 proteins. In summary, the structure of Orf63 presents a newly identified fold and mode of dimerization that is exclusive to the λ family of bacteriophages.

The Cα RMSD between one protomer of the NMR structure and the AlphaFold model was 1.80 Å. A graphical comparison is presented in Supplementary Fig. S9. The deviation between the NMR structure and AlphaFold model is attributed a difference in the angle between the two helices, α1 and α2 (16° in the NMR structure and 32° in the AlphaFold model). In addition, the AlphaFold model predicted longer helices (α1: 6–22; α2: 34–62) than what was observed in the NMR structure.

**Figure 3:**
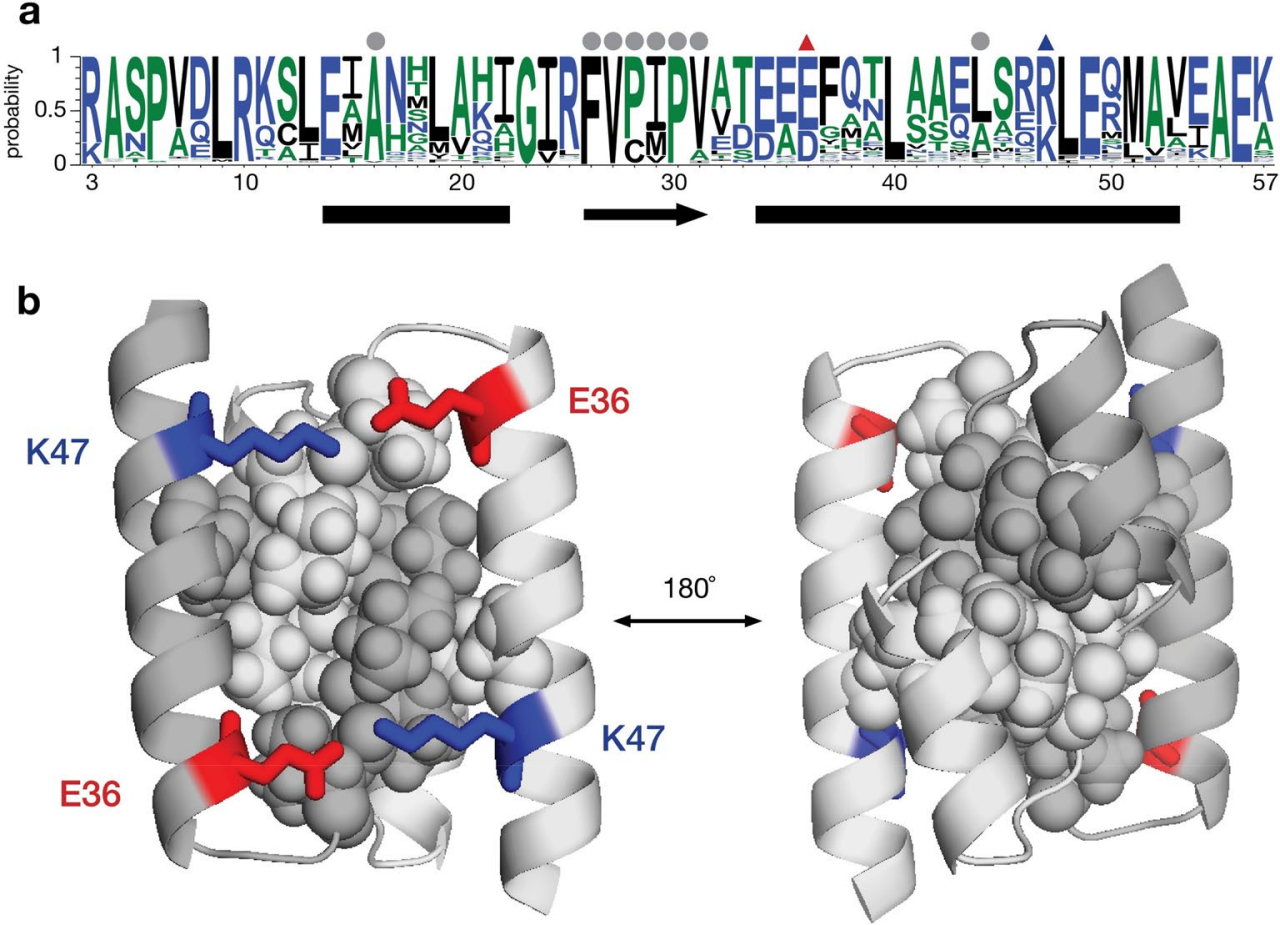
Sequence variation among Orf63 protein in the UniRef100 database. (**a**) Above the WebLogo consensus plot, a dot at nonpolar (orange), acidic (red), and basic positions (blue) indicates conservation > 80%. Triangles indicate positions where an ionic bond was observed in the refined structure. (**b**) Cartoon and space filling views of the most conserved residues with the indicated ionic bonds.

Variation among a set of 290 Orf63 homologs in the UniRef100 database is presented in Fig. 3. As expected, most of the conserved amino acids mapped to the hydrophobic core of the protein. The central β-strand bridge was intolerant to variation at positions corresponding to F28, V29, and P32 in λ Orf63. Two arginines (R3/R10) that located in the unstructured N-terminal region appear to be conserved with unknown significance. Within the folded region, E36 and K47 stand out as two well conserved charged amino acids. The refined NMR structures suggest that they have the potential to form an ionic bond.

**Figure 4:**
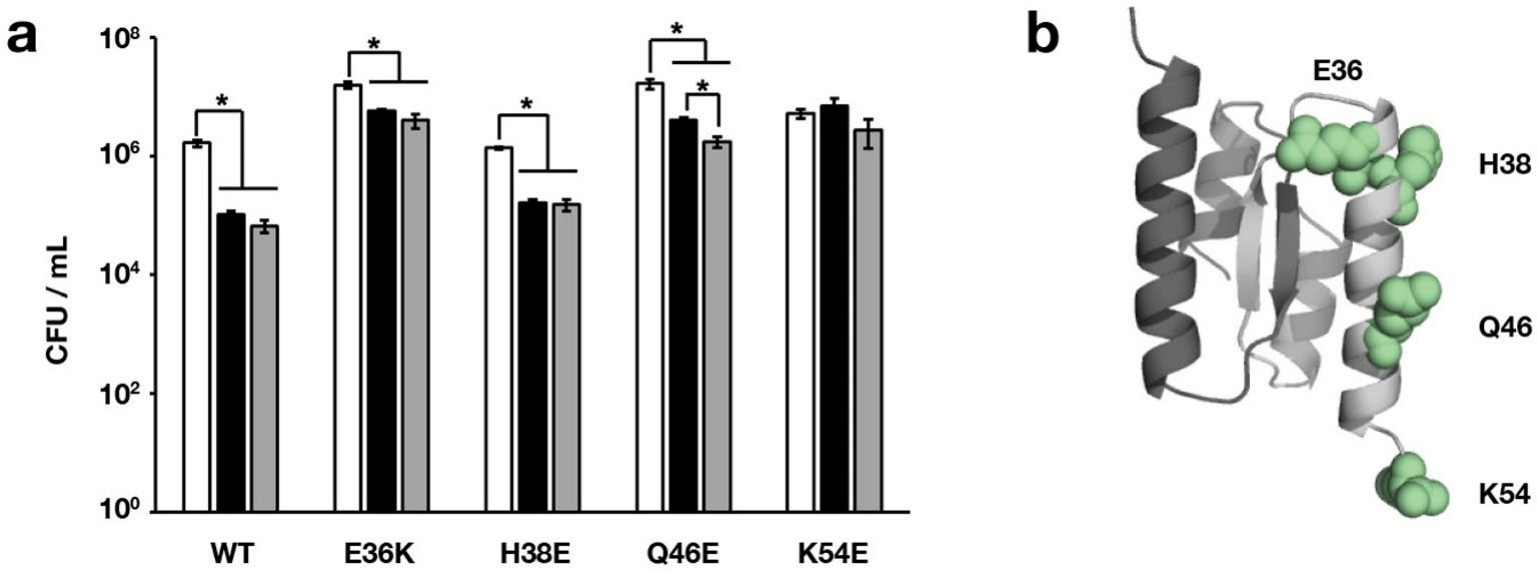
Effect of Orf63 and four variants on host survival and viral development. (**a**) Phage λΔ*orf63* at an m.o.i. of 5 was added to *E. coli* C600 previously induced with IPTG to express different variants of the *orf63* gene and to produce different forms of the Orf63 protein. White bars represent bacteria that were mock infected with buffer as a negative control. Black bars represent bacterial survivors post-infection. Grey bars represent lysogens among bacterial survivors. All counts are expressed as the number of colony forming units (CFU) per mL. Errors are presented as SD from three replicates. (**b**) Location of the variants examined on the structure of Orf63.

*Escherichia coli* expressing either wild type Orf63 or one of four substitutions at surface exposed residues in helix α2 demonstrated different degrees of survival when infected with λ phage bearing a deletion in the *orf63* gene. While not conserved among all Orf63 homologs, they nevertheless offer a first opportunity to probe biological function. Each of white bars in Fig. 4 represent a mock infection in each assay varying from 10^6^–10^7^ CFU/mL. When the activity of *orf63* is restored, the number of bacterial survivors and lysogens is reduced at least ten-fold. A similar reduction in survivors and lysogens was observed for Orf63[H38E] and Orf63[Q46E]. In contrast, Orf63[K54E] did not enhance the lytic developmental program. K54 is in an unstructured region beyond the boundary of helix α2 that may provide a necessary contact a host nucleic acid or protein. Curiously, in the sequence comparsion, glutamic acid is the predominating amino acid at this position and lysine only makes a minor contribution leading to the possiblity that not all Orf63 proteins in λ family bacteriphages are active. Orf63[E36K] did not reduce bacterial survival and lysogen production to the same extent at the expressed wild type Orf63 protein suggesting this variant may also be defective.

**Figure 5:**
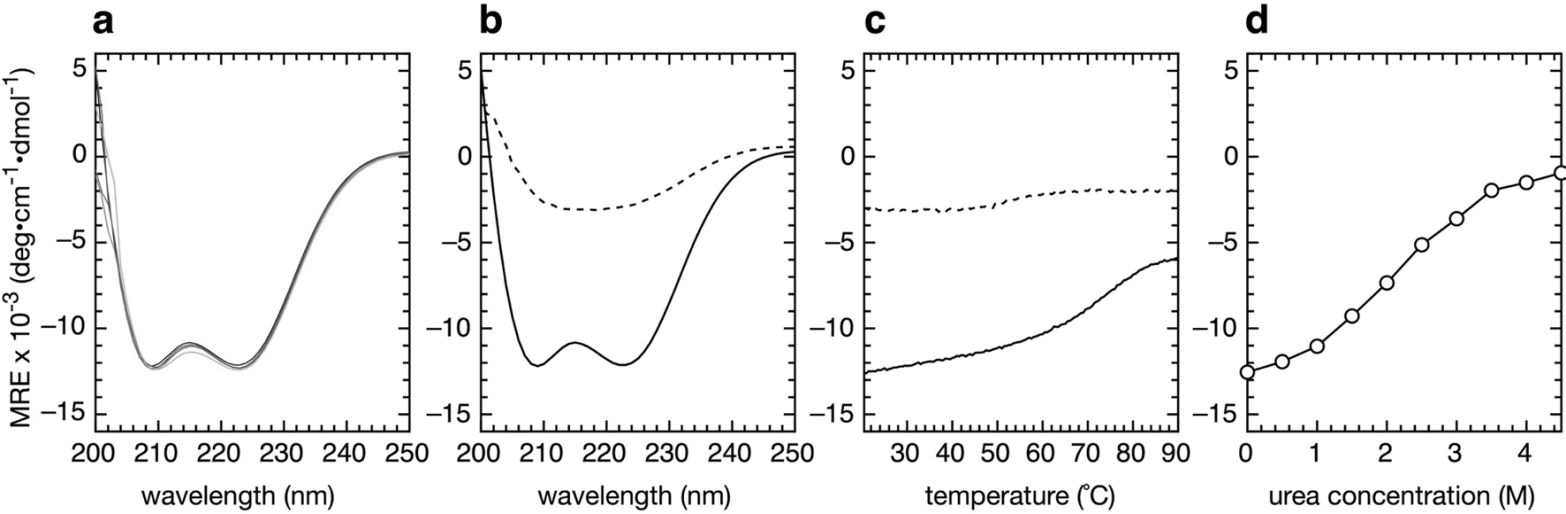
CD spectroscopy. (**a**) Spectra of wild type Orf63 with varying concentration of NaCl (black: 0 M; red: 0.5 M; cyan: 1.0 M; green: 2.0 M; magenta: 4.0 M). The base buffer for each assay is 10 mM Tris–HCl, pH 7.4. (**b**) Far UV spectrum of wild type Orf63 (solid line) and Orf63[E36K] (dashed line). (**c**) Thermal denaturation of Orf63 (solid line) and Orf63[E36K] (dashed line) monitored at 222 nm. (**d**) Urea denaturation of wild type Orf63 (circles).

A prior study performed on lysozyme demonstrated that salt bridges with an accessible/buried surface area ratio (ASA) greater than 50% did not contribute to the stability of the protein when the assay buffer contained as little as 0.2 M KCl^19^. Given that the ASA of E36 is 77% (Supplementary Table 2), a potential E36/K47 salt bridge in Orf63 would be predicted to contribute very little to the stability of Orf63. Indeed, far-UV circular dichroism (CD) spectra of Orf63 in buffers containing up to 4 M NaCl had no effect on the secondary structure of the Orf63 (Fig. 5a). Expression and the subsequent study of purified preparations of Orf63[E36K] protein, however, showed that flipping the charge was highly destabilizing to the extent that Orf63[E36K] appeared to be unfolded. Initial evidence for a folding defect in Orf63[E36K] was observed during protein expression since the culture temperature had to be reduced from 37 to 30 °C to produce soluble protein. While CD spectra of wild type Orf63 demonstrated typical α-helical signals at 208 and 222 nm, Orf63[E36K] CD spectra strongly resembled the spectra of an unfolded protein (Fig. 5b). A subsequent thermal denaturation assay of wild type Orf63 monitored at 222 nm revealed a strong transition with a T_m_ of 75° and a slight transition at 54 °C for Orf63[E36K] that may not be significant (Fig. 5c). Orf63 was moderately resistant to urea denaturation with a transition at a concentration of ~ 2.5 M (Fig. 5d). Chemical and thermal denaturation of wild type Orf63 was reversible.

A bioinformatics survey that focused upon a set of domains of unknown functions (DUFs) suggested that Orf63 may have nucleic acid binding properties^20^. Building upon our observation that polar, solvent facing Orf63 variants affected activity, a sample of ^15^N-Orf63 with mixed with a molar excess of a 32 bp palindromic dsDNA. A comparison of ^1^H-^15^N HSQC spectra of free and DNA-bound Orf63 showed no chemical shift changes or line broadening suggesting that in this limited context, Orf63 may require a specific DNA sequence to achieve high affinity binding or not bind dsDNA, at all.

**Figure 6:**
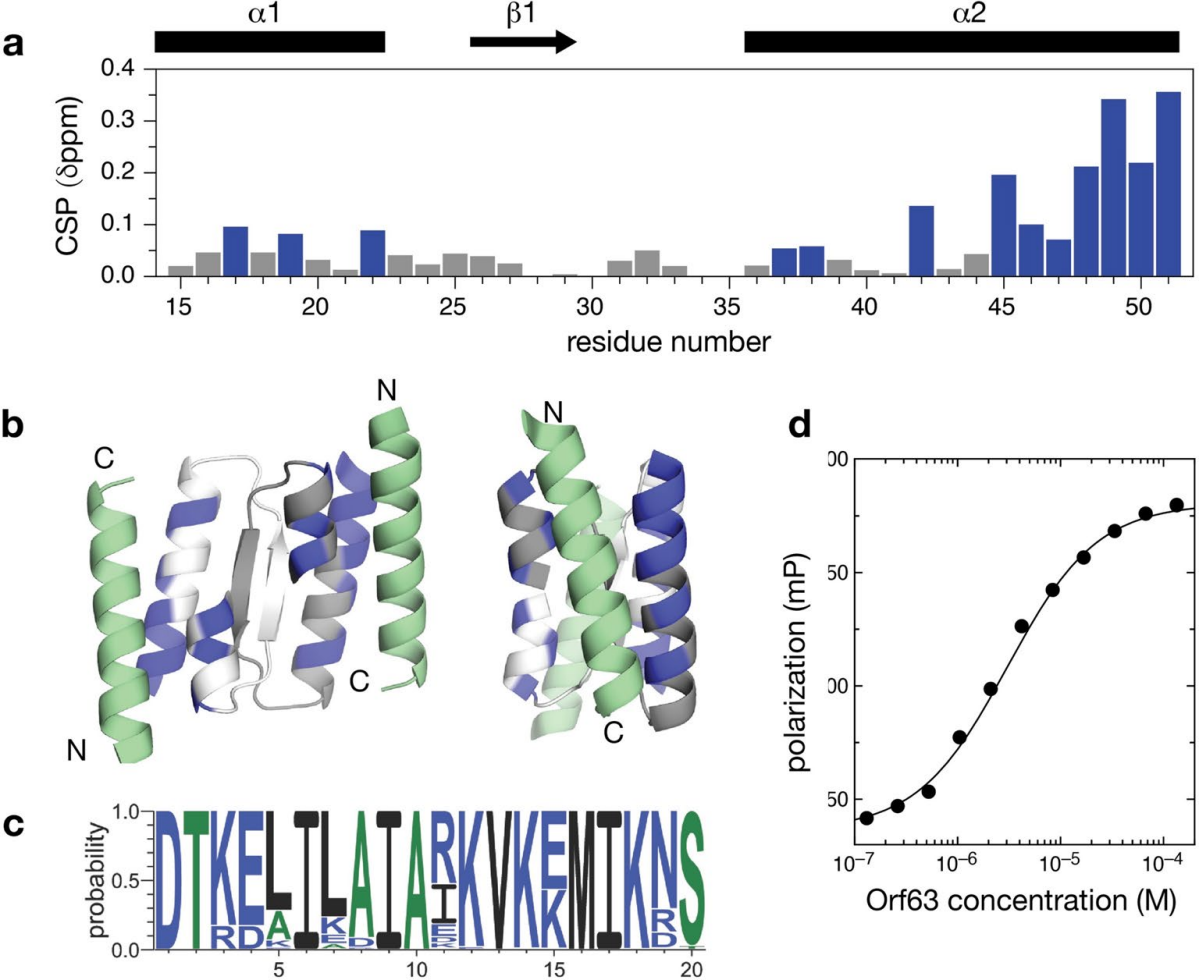
A designed α-helix binds a cleft made by helix α1 and α2 of Orf63. (**a**) ^1^H-^15^N chemical shift perturbations (CSPs) measured between ^15^N-Orf63 free, and in complex with a unlabelled designed peptide. Blue shading highlights a CSP > 0.05. (**b**) Two views of the designed helix (green) docked to the Orf63 dimer (white, grey) again with a CSP > 0.05 in blue. The N- and C-termini of the Orf63 and the designed peptide are shown for orientation. (**c**) Sequence variation throughout the designed helix sampled by Rosetta (**d**) Fluoresence polarization changes observed in mixtures containing a 10 nM designed peptide and varying concentrations of Orf63 (expressed in terms of one protomer to reflect the predicted 1:1 peptide:protomer ratio).

Consequently, if Orf63 has the potential to bind another protein, a hydrophobic cleft formed between helix α1 and α2 emerges an interesting candidate to explore (Fig. 6). Using a machine learning approach based on AlphaFold, a single helix was identified that made complementary contacts to Orf63. The helical candidate from this approach was further refined with Rosetta to reveal the most important positions on the ligand and how much they could be varied. From a sequence comparison of the lowest energy sequences in the Rosetta refined ensemble, a 20-mer peptide candidate was identified and synthesized for biochemical studies. The molecular model of peptide candidate was predicted to make complementary hydrophobic contacts to the cleft supported by two peripheral ionic contacts. From a fluorescence polarization assay, the affinity of the designed peptide was determined to be 3.1 ± 0.3 µM under the assumptions that there was a 2:1 binding ratio of peptide and Orf63 dimer and the two peptide binding sites were independent. Since the N-terminus of the helical ligand was modeled to be near the C-terminus of Orf63, a hybrid protein was designed consisting of Orf63 followed by an 11 amino acid linker and the helical peptide. The ^1^H,^15^N-HSQC spectrum of the hybrid protein resembled Orf63 with additional peaks from the helical ligand along with a set of chemical shift changes likely arising from structural differences at the Orf63-ligand interface (Supplementary Fig. S10). To confirm the specificity of the interaction, the same 20-mer sequence was expressed as a 6xHis-tagged-Ubquitin fusion protein. A 2:1 mixture of the Ubiquitin fusion protein and ^15^N-labelled Orf63 dimer revealed significant line broadening suggesting binding was occurring (total molecular weight of the complex was 45 kDa). In contrast, a 2:1 mixture of a Ubiquitin fusion protein with an unrelated sequence produced no chemical shift or line broadening changes (Supplementary Fig. S11). Finally, a synthetic peptide was titrated into ^15^N-labeled Orf63 to perform a chemical shift perturbation (CSP) mapping study. Since there were no spectral changes observed between a 1:1 and a 1.2:1 peptide:protomer ratio, the complex was deemed to be saturated with peptide ligand. The CSPs observed could be divided into a majority of resonances in fast exchange and a minority of resonances in helix α2 from S45 onwards in slow exchange. The resonances in slow exhange also had the largest measured CSPs suggesting that the latter part of helix α2 were participating in ligand binding the most. When all CSPs were mapped on the structure of Orf63, the observed interaction surface was consistent with the predicted interaction surface of the model.

## Discussion

Orf63 is a non-essential dimeric protein in both λ and related Shigatoxigenic phages that promotes lytic development over lysogenic development. The *orf63* gene is located within a cluster of conserved, yet under-characterized open reading frames found in the *exo-xis* region of the phage genome. From sequence and structural data mining, Orf63 is unique to temperate phages. Despite its small size, it is not observed as a domain in any other protein.

The NMR structure of Orf63 reveals a unique fold with a dimer interface drawing contributions from each of the two α-helices and one β-strand. The structure refinement suggested a potential ionic bond between E36 and K47 across the dimer interface. Evidence against the significance of a E46/K47 ionic interaction came from its predominatly solvent exposed position and insensitivity of Orf63 to high ionic strength. The charge substituted variant Orf63[E36K] was unfolded by CD spectroscopy and consequently inactive in a biological assay that measured two attributes of a vigorous lytic developmental response. If a salt bridge is conserved but not important, the drastic effect of the E36K substitution perhaps may be explained by formation of new ionic interactions with local amino acids such as E32 in λ Orf63 or by charge repulsion with K47.

Approximate one-third of λ Orf63 consists of unstructured segments that flank central α1β1α2 domain. Given the sequence conservation several amino acids in these unstructured segments, it is tempting to speculate that they may be involved in protein networks by donating small linear motifs^21^ to a protein partner. A mass spectrometry-based proteomics survey is an obvious experimental route toward identifying Orf63 protein partners either from viral or bacterial origins. It still remains that Orf63 could be a RNA binding protein since RNA was not tested as a potential ligand.

In the absence of established binding partners either of viral or host origin, a machine learning method was used to identify a minimal motif that could interact with the cleft. The prevailing solution was an amphipathic helix with micromolar affinity that made complementary hydrophobic contacts with the cleft and peripheral ionic contacts. If one helix is sufficient to support an interaction with Orf63, it open the potential for a variety of host and viral proteins to partner with Orf63. After this study was performed, the machine learning software RFdiffusion was released enabling larger binding proteins to be designed^22^. When the RFdiffusion method was configured to create a small protein partner of 80–100 amino acids, the same short helical ligand was repeatedly observed in the output thereby reinforcing the initial observation that one helix would be sufficient. The ability to design antagonistic binding peptides and proteins offers against phage developmental proteins may offer a new way to create therapeutics against EHEC outbreaks.

## Methods

### Expression and purification of proteins

Phage λ *orf63* (laboratory reference #201916, UniProt: Q38267; DUF1382) was gene synthesized by ATUM (Menlo Park, CA) with an amino-terminal 6xHis + FLAG tag and inserted into plasmid pD441NH (T5 promoter). Amino acid substitution mutants were prepared using the Q5 mutagenesis method (New England Biolabs) with oligonucleotides from IDT. Sequencing was performed by The Centre for Applied Genomics (Hospital for Sick Children; Toronto, ON) Expression was achieved in cultures under 50 µg/mL kanamycin selection at 30 °C in an *E. coli* BL21. At an A600 of 0.8, expression was induced by the addition of 1 mM isopropyl thiogalactoside (IPTG), and the culture was grown for 3 h further before harvesting. Detailed information regarding the purification of this fragment by nickel affinity and gel filtration chromatography has been published previously^13^. For NMR spectroscopy, a uniformly ^13^C, ^15^N isotopically labelled protein sample was made from a 1 L culture containing M9 minimal media salts supplemented with 3 g of ^13^C-glucose (Sigma-Aldrich), 1 g of ^15^N ammonium chloride (Sigma-Aldrich) and 1 g of ^13^C,^15^N Celtone algal extract (Cambridge Isotopes). Natural abundance Orf63 proteins were prepared using a similar protocol except cultures were grown in LB media. Protein concentrations were estimated by UV absorbance at 280 nm. Two ubiquitin fusion proteins with an N-terminal 6xHis tag, linker GGLVPRGSG, and either the sequence DTKELILAIARKVKEMIKNS corresponding to the designed Orf63 binding peptide (pET28-UbqOrf63L2; reference #7A03005 or an unrelated peptide MAIAHAATEYVFADFVLK (pET28-UbqPanxL1; reference #7A03212-2) were expressed in E. coli BL21:DE3 at 37 °C, and purified according to the two step nickel-affinity / gel-filtration protocol. The final buffer for all expressed protein was either NMR bufffer (5 mM Tris–HCl pH 7.4, 50 mM NaCl, 0.05% NaN_3_) or phosphate buffered saline (PBS) pH 7.4. A hybrid protein consisting of an N-terminal 6xHis tag, Flag-tag, the Orf63 sequence, a linker GSGSLVRGSGA, and the sequence DTKELILAIARKVKEMIKNS was similarily expressed and purified. 

### Fluorescent peptide binding assay

Fluorescence polarization of a series of 100 uL samples containing a 10 nM fluorescent peptide (FAM-Ahx-DTKELILAIARKVKEMIKNS) manufactured by LifeTein (Somerset, NJ) and varying concentrations of Orf63 in phosphate buffered saline buffer pH 7.4 (Gibco) were measured in a Synergy H4 plate 96-well plate reader (Agilent) with standard parameters (ex: 495 nm, em: 520 nm). Each measurement was made in triplicate and averaged. Affinity was determined by fitting the points to a 1:1 binding function assuming each peptide bound each monomeric unit of Orf63 independently and the difference in polarization was negligible whether one or two 2.7 kDa peptides were bound the 18 kDa Orf63 protein dimer.

### NMR sample preparation

A preparation of λ Orf63 protein was concentrated to 0.8 mM in NMR buffer supplemented with 10% (v/v) D_2_O for NMR spectroscopy. For NMR experiments requiring a sample in D_2_O, the Orf63 protein was dialyzed to a similar buffer in 98% (*v/v*) D_2_O. A mixed ^12^C/^13^C sample was made by mixing ^12^C/^14^N and ^13^C/^15^N proteins at a 1:1 molar ratio, adding urea to 6 M and rapidly diluting the mixture into a 20-fold excess of NMR buffer. The protein was concentrated and dialyzed to a 98% (*v/v*) D_2_O buffer.

### NMR spectroscopy for structure determination

A series of 2D and 3D heteronuclear NMR spectra were acquired at a temperature of 308 K on a 700 MHz Bruker AvanceIII spectrometer equipped with a nitrogen-chilled probe to support the backbone assignments that had been previously described. These experiments included (2D-^15^N-HSQC, 2D-^13^C-HSQC, 3D-CCONH, 3D-HCCONH, 3D-HBHACONH, and 3D-CCH-TOCSY). The 3D experiments were acquired according to a 10–20% sparse sampling schedule and processed with NMRPipe^23^ and HMSist^24^. Distance restraints were obtained from a 3D-^15^N-NOESY and a 3D-^13^C-NOESY experiments sparsely sampled at 20%. Intermolecular distance restraints were obtained from a 3D-^12^C-filtered/^13^C-separated NOESY experiment^26^ acquired at the laboratory of Lewis Kay (Univ. Toronto) on a Varian Inova 600 MHz instrument equipped with a room temperature probe.

### NMR spectroscopy to assess peptide and nucleic acid binding

A titration was performed by acquiring a series of ^15^N-HSQC spectra of 0.48 mM ^15^N-Orf63 (monomeric concentration) with 0.12, 0.24, 0.36, 0.48, and 0.60 mM peptide (DTKELILAIARKVKEMIKNS) manufactured by GenScript (Piscataway, NJ) in phosphate buffered saline pH 7.4. Assignments of peptide-bound ^15^N-Orf63, particularly through two helical regions, were made by walking through HN-HN correlations in a 3D 15N-NOESY spectrum. Weighted chemical shift differences (*d*) between unbound Orf63 and peptide bound Orf63 were determined using the equation $d = \sqrt {(0.5 \cdot \left( {\delta_{H}^{2} + \left[ {0.14 \cdot \delta_{N}^{2} } \right]} \right)}$. A comparison was also made between ^15^N-HSQC spectra of ^15^N-labeled 6xHis-Flag-Orf63 (at 0.15 mM) and a ^15^N-labeled 6xHis-Flag-Orf63-linker-peptide hybrid protein (also at 0.15 mM). Nucleic acid binding was determined by mixing ^15^N-labeled 6xHis-Flag-Orf63 at 0.1 mM (monomeric concentration) with 0.2 mM Sox9 palindromic dsDNA that had previously been annealed^19^.

### Structure determination

NMR spectra were analyzed with CCPN Analysis 2.52^25^. Backbone dihedral angles were predicted from chemical shifts with TALOS^26^. An initial ensemble of 100 structures sorted by the lowest number of NOE violations were calculated from a set of 10,000 structures using CYANA 3^27^. The ensemble was refined using Rosetta with distance, angle and hydrogen bond restraints converted to Rosetta .cst format. Symmetry was enforced throughout the calculation. Details of the Rosetta refinement method have been published^28^. The top 20 refined structure solutions that satisfied the experimental restraints the most formed the final ensemble. Structural quality was assessed with PSVS^29^ and PROCHECK^30^.

### Determination of oligomeric state

Size-exclusion chromatography multi-angle laser scattering (SEC-MALS) was performed at the Hospital for Sick Children SPARC analytical facility (Toronto, ON) on a 1260 Infinity-II HPLC (Agilent) and SEC300A 2.7 µM 4.6 × 300 mm column (Advance Bio) analytical column at 0.2 mL/min linked to MiniDawn TREOS MALS and a OptiLab T-rEX refractive index detection instruments (Wyatt). The chromatography and detection system were equilibrated in 10 mM Tris–HCl, 0.15 M NaCl, pH 8.0 for 18 h before the first detection. A preliminary injection of 2 mg/mL of bovine serum albumin was performed to calibrate peak and retention time profiles of the detectors prior to sample analysis. For the primary Orf63 analysis, a 2 mg/mL sample was centrifuged at 13,000 *g* at 4 °C for 10 min to remove any precipitates and other large, insoluble particles and applied to the system. Chromatogram data was analyzed with ASTRA (Wyatt) with a *dn/dc* of 0.185.

### Bioinformatics and structure predictions

The UniRef100 database^31^ was searched with mmseqs2^32^ for homologs to λ Orf63. Any truncated sequences were removed. The dataset was realigned in AliView^33^ and exported as FASTA formatted list to WebLogo3^34^. AlphaFold Multimer was used to predict the dimeric structure of Orf63 (https://github.com/sokrypton/ColabFold).

### Binding protein design

AfDesign was used to predict five 20-residue binding peptides for Orf63 (https://colab.research.google.com/githum/sokrypton/ColabDesign/blob/* main/af/examples/peptide_binder_design.ipynb).* To assist modeling, a binding site on Orf63 was declared as a set of hotpot residues. The best design docked to Orf63 was used as input to Rosetta *fixbb* design with all amino acid positions being allowed to vary. From an ensemble of 512 structures, a consensus sequence was determined. The sequence with the highest number of observations was selected. A hybrid Orf63 protein was designed by appending the sequence of best design to the C-terminus of Orf63 separated by the linker, GSGSLVPRGSGA. A codon optimized gene corresponding to this design was synthesized by Gene Universal (Newark, DE) and inserted into pET29b. Expression and purification were achieved by growing a culture in M9 media for ^15^N-labeling at 37 °C and following the previously described protocol for induction of gene expression and protein purification.

### Bacterial survival assay

To stimulate the production of Orf63 protein or its derivatives from the pD441NH expression plasmid, overnight bacterial cultures of *E. coli* C600 were diluted 100-fold in a fresh LB medium and treated with 1mM IPTG. Host bacteria were cultivated at 37 °C to A600 of 0.1. A 0.1 mL aliquot was centrifuged (2000 *g* for 10 min at 4 °C). and afterwards, the pellet was washed with TCM buffer (10 mM Tris–HCl, 10 mM MgSO4, 10 mM CaCl_2_, pH 7.2) twice, and then resuspended in the same buffer. Bacteriophage λΔ*orf63* was added to bacteria at an m.o.i. of 5. The mixture was incubated at 37°C for 15 min and afterwards, serial dilutions in 0.85% NaCl were prepared. From these dilutions, 40 μL of each suspension was spread on LB agar plates supplemented with 25 µg/mL kanamycin. After an overnight incubation at 37 °C, the number of *E. coli* survivors per mL (CFU/ml) was counted.

### Lysogenic conversion of survivors

Fresh bacterial colonies from an appropriate dilution were placed in a 96-well plate filled with 200 μL of LB medium and shaken at 37 °C until the cultures reached an A600 of 0.1. The cultures were then treated with 50 J/m^2^ UV light to induce phage production and incubated at 37 °C for 2 h further. The lysogens in the sample were mixed with chloroform and centrifuged a 2000 *g* for 10 min at 4 °C. The aqueous phase was spotted onto double-layer LB agar. After overnight incubation at 37 °C, the number of lysogens on each agar plate was determined.

### Supplementary Information

Supplementary Information.

## Data Availability

Chemical shifts of λ Orf63 were deposited in the BMRB (entry 51519; bmrb.io/data_library/summary/index.php?bmrbId = 51,519). The final ensemble of structures was deposited in the PDB (entry 8DSB; www.rcsb.org/structure/8DSB).
