# Supplementary material for: The NMR structure of the Orf63 lytic developmental protein from lambda bacteriophage
Source: Sci Rep. 2024 Feb 15;14:3793. doi: 10.1038/s41598-024-54508-9 (PMC10869804; doi:10.1038/s41598-024-54508-9)
Supplement: Supplementary file 1 — Supplementary Information. [file 41598_2024_54508_MOESM1_ESM.pdf]

Supplementary Information

The NMR structure of the Orf63 lytic developmental protein from bacteriophage lambda

Naushaba Khan[1], Tavawn Graham[1], Katarzyna Franciszkiewicz[2], Sylwia Bloch[2], Bożena Nejman-Falenczyk[2], Alicia Wegrzyn[2], and Logan W Donaldson[1*]

[1]York University, Department of Biology, Toronto, ON, M3J1P3, Canada
[2]University of Gdańsk, Department of Molecular Biology, Gdańsk, 80-308, Poland
*e-mail: logand@yorku.ca

Table S1. Structural statistics for the Orf63 NMR structure

NOE distance restraints in the ensemble[a]	796
intraresidue	373
short ($\|i\text{-}j\| = 1$)	163
medium ($1 \leq \|i\text{-}j\| \leq 5$)	73
long ($\|i\text{-}j\| > 5$)	113
interchain	74
Hydrogen bond distance restraints	
HN–O / N–O pairs	25
Torsion angle restraints	
backbone (Φ / Ψ)	30
Structural quality analysis	
close contacts	0
RMS deviation of bond angles (deg)	0.3
RMS deviation of bond lengths (Å)	0.0009
RMS deviation to the mean coordinates[b]	
all backbone / heavy atoms (Å)	0.8 / 1.1
Ordered backbone / heavy atoms (Å)	0.5 / 0.8
Ramachandran plot[c] (%)	
residues in most favored regions	98.8
residues in additional allowed regions	1.2
residues in generously allowed regions	0.0
residues in disallowed regions	0.0

[a] None of the twenty structures in the ensemble (PDB: 8DSB) has a distance violation > 0.2 Å and a dihedral angle violation > 5°.

[b] Ordered residues (16-54) are defined by a dihedral angle order parameter with S(Φ)+S(Ψ)>=1.8 as determined by PSVS.

[c] Determined by PROCHECK.

Table S2 — Intermolecular interface analysis. Output from PISA (Krissinel & Henrick 2007; ASA; accessible surface area in Å^2; BSA, buried surface area in Å^2). Of the 43 residues that comprise the folded Orf63 protomer with an total ASA of 4109 Å^2, 27 residues participate in the binding interface with a total BSA of 1215 Å^2. Residues with a BSA/ASA ratio > 80% are shaded.

residue	ASA	BSA	BSA/ASA (%)
12 SER	136.9	17.2	13
13 ILE	100.1	76.8	77
14 GLU	148.1	0.0	0
15 MET	89.8	24.1	27
**16 ALA**	**22.5**	**22.3**	**99**
17 HIS	85.0	39.5	46
18 SER	55.5	0.0	0
19 LEU	12.6	7.0	56
20 ALA	64.2	19.3	30
21 GLN	151.8	0.0	0
22 ILE	114.9	0.0	0
23 GLY	69.5	0.0	0
24 ILE	30.0	4.9	16
25 ARG	232.3	127.1	55
**26 PHE**	**74.7**	**64.7**	**87**
**27 VAL**	**112.6**	**112.6**	**100**
**28 PRO**	**108.1**	**108.1**	**100**
**29 ILE**	**108.8**	**88.2**	**81**
**30 PRO**	**109.1**	**109.1**	**100**
**31 VAL**	**57.3**	**47.5**	**83**
32 GLU	169.4	45.1	27
33 THR	71.1	1.1	2
34 ASP	111.3	23.8	21
35 GLU	130.4	0.0	0
36 GLU	71.3	16.7	23
37 PHE	91.3	52.5	57
38 HIS	126.4	0.0	0
39 THR	89.4	0.0	0
40 LEU	69.3	32.1	46
41 ALA	30.0	4.9	16
42 ALA	46.6	0.0	0
43 SER	48.1	3.1	6
**44 LEU**	**56.7**	**48.6**	**86**
45 SER	59.0	0.0	0
46 GLN	110.3	0.0	0
47 LYS	128.6	78.3	61
48 LEU	44.0	3.9	9
49 GLU	114.7	0.0	0
50 MET	138.9	0.0	0
51 MET	107.1	50.4	47
52 VAL	95.8	0.0	0
53 ALA	71.4	0.0	0
54 LYS	244.7	0.0	0

a

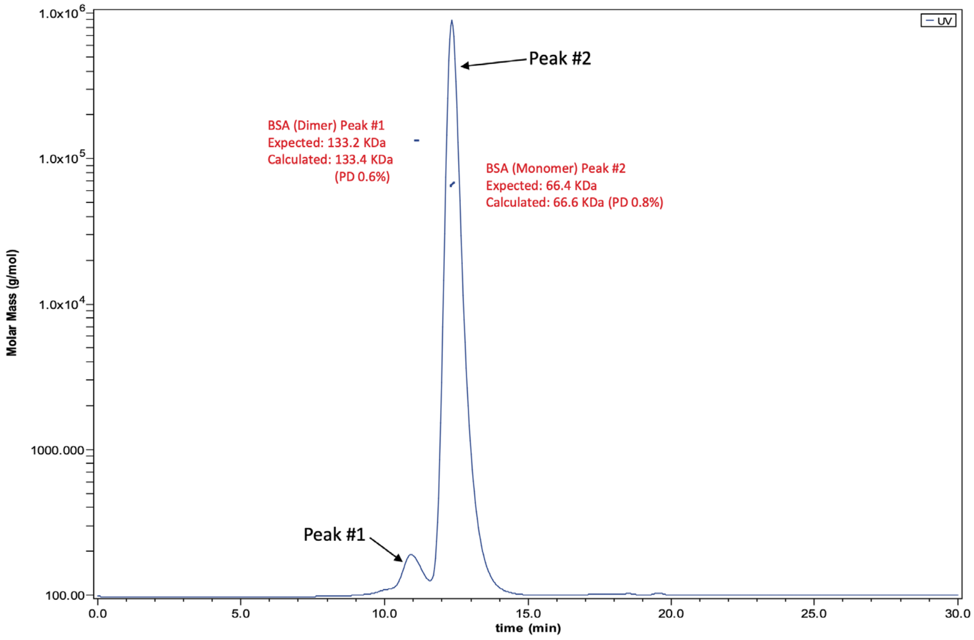

b

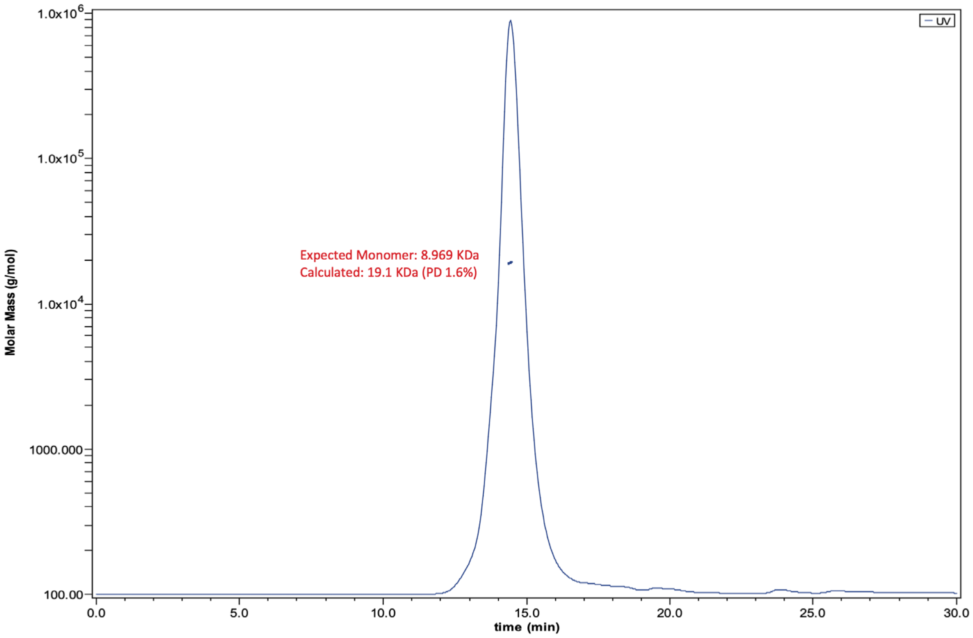

Figure S1 — Size exclusion chromatography / multi-angle laser scattering analysis (SEC-MALS). **(a)** BSA control for instrument calibration. **(b)** A 2 mg/mL injection of λ Orf63. Analysis of the primary peak demonstrates that the protein is dimeric in solution.

```
         1         2         3         4         5         6         7
12345678901234567890123456789012345678901234567890123456789012345678901234567 PDB 8DSB / as expressed
HHHHHHMDYKDDDDKHKASSVELRTSIEMAHSLAQIGIRFVPIPVETDEEFHTLAASLSQKLEMMVAKAEADERNQV
              123456789012345678901234567890123456789012345678901234567890123 native sequence
                       1         2         3         4         5         6
```

Figure S2 — The PDB coordinate file is transposed by 14 amino acids relative to the native Orf63 sequence due to an N-terminal 6xHis (green) and FLAG tag (blue). For example, refer to E36 in the paper as E50 in the PDB coordinate file.

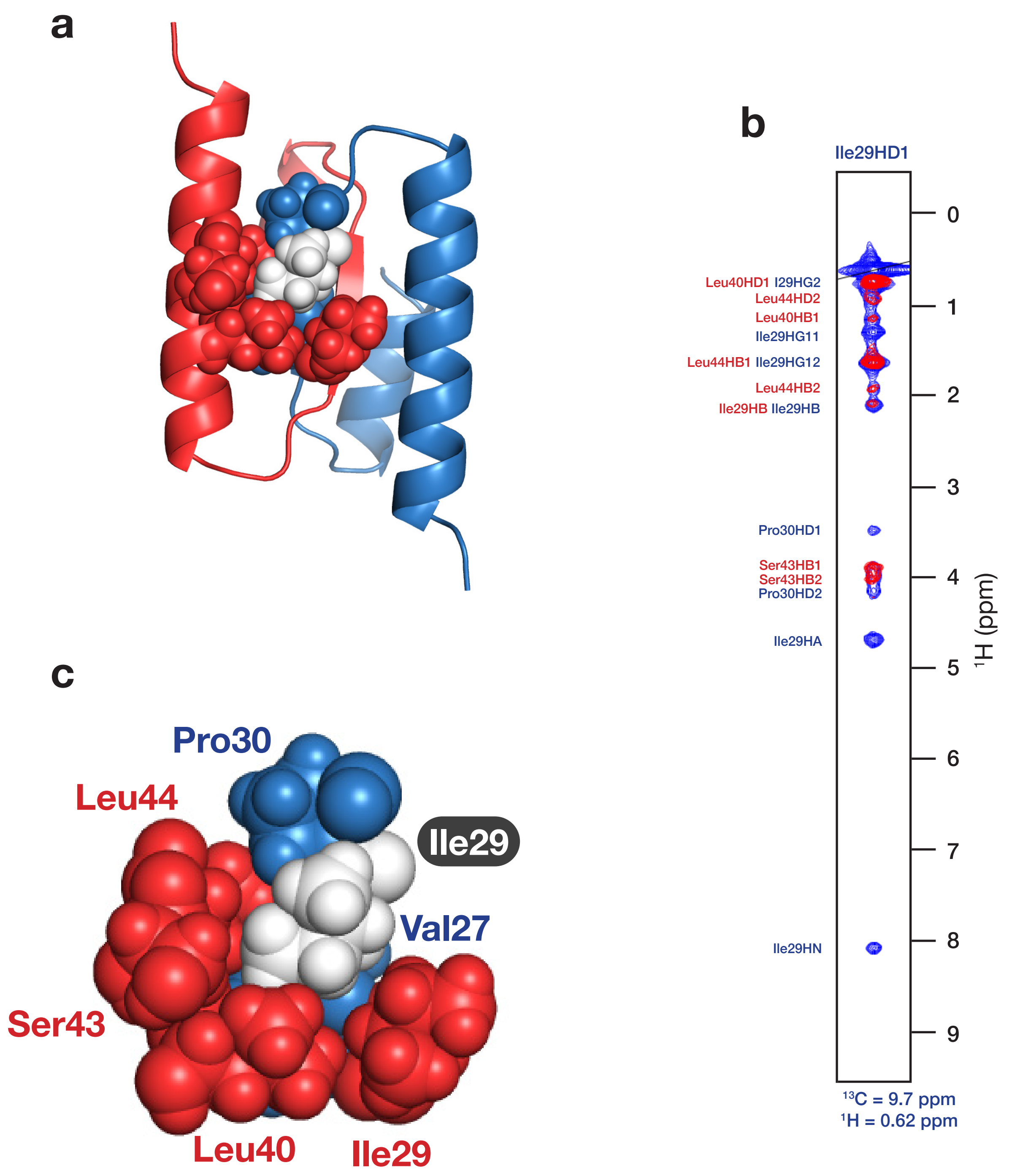

Figure S3 — NOE observations supporting the structure of Orf63. **(a,b)** Cartoon diagram and magnification of intramolecular amino acids (blue) and intermolecular amino acids (red) in the vicinity of Ile29 (white). **(c)** A plane from a 3D ^{13}C-edited NOESY spectrum (blue) and 3D ^{12}C-filtered,^{13}C-edited NOESY spectrum (red) highlighting NOEs from the HD1 methyl group of Ile29.

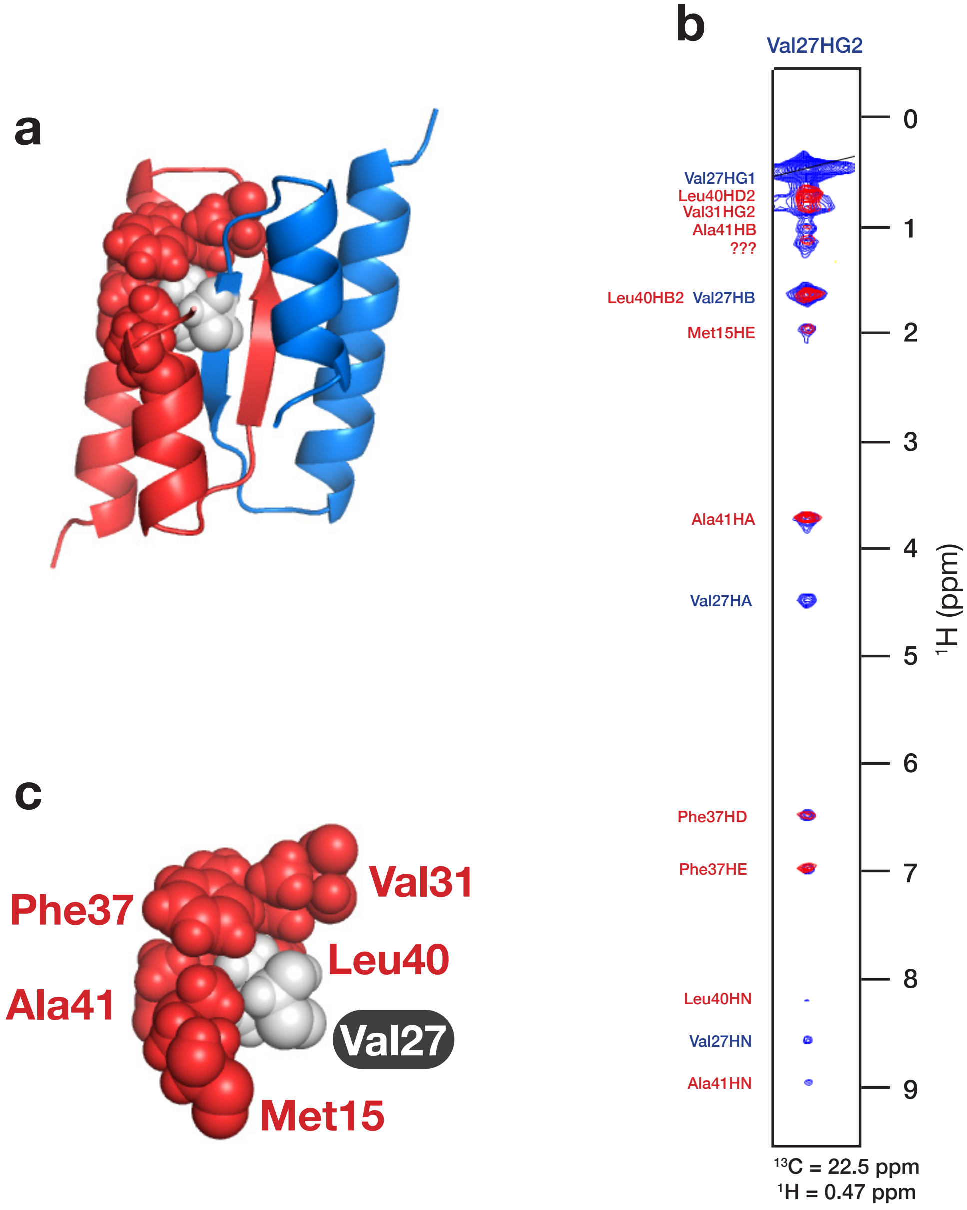

Figure S4 — NOE observations supporting the structure of Orf63. **(a,b)** Cartoon diagram and magnification of intramolecular amino acids (blue) and intermolecular amino acids (red) in the vicinity of Val27 (white). **(c)** A plane from a 3D ^{13}C-edited NOESY spectrum (blue) and 3D ^{12}C-filtered,^{13}C-edited NOESY spectrum (red) highlighting NOEs from the HG2 methyl group of Val27.

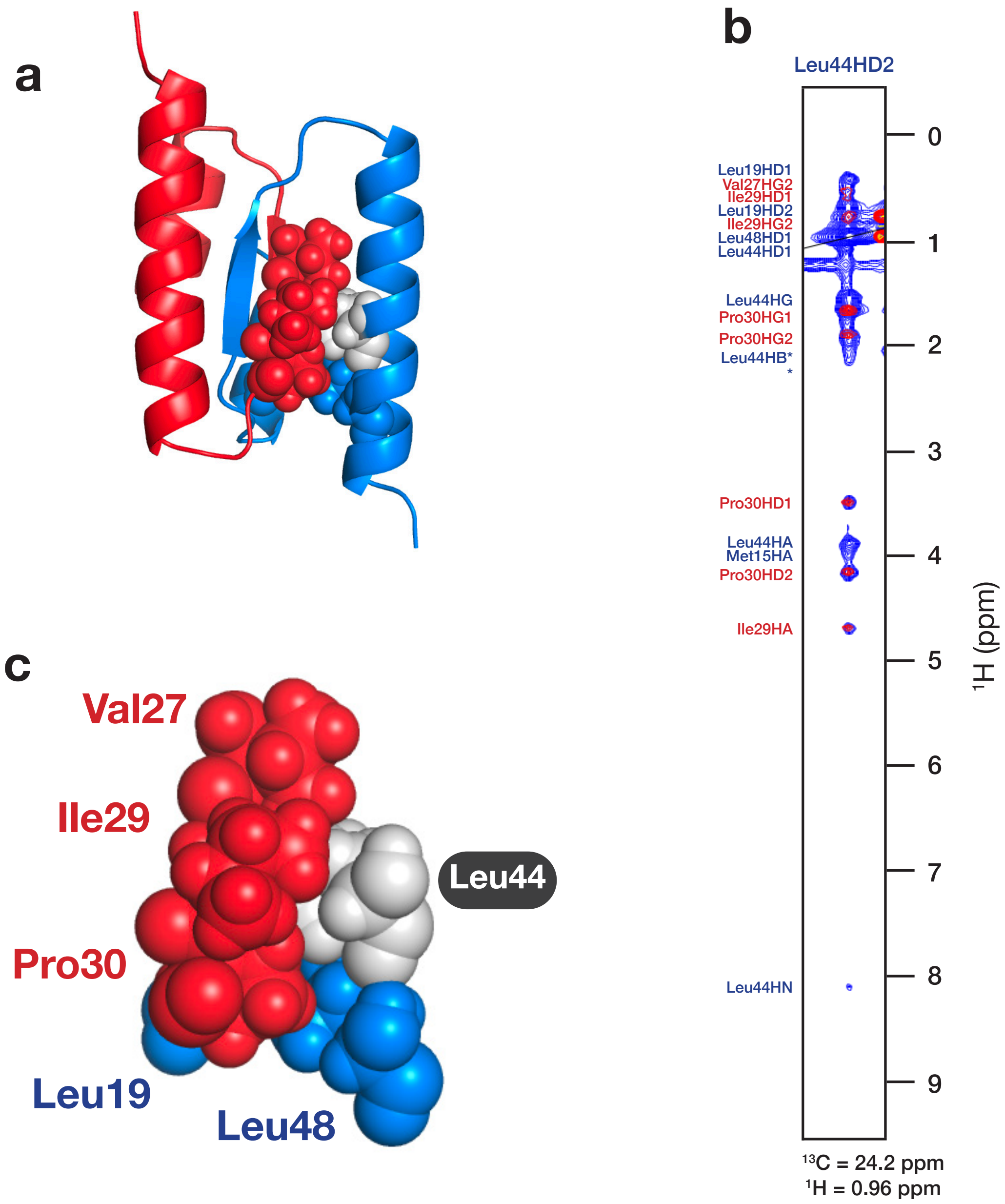

Figure S5 — NOE observations supporting the structure of Orf63. **(a,b)** Cartoon diagram and magnification of intramolecular amino acids (blue) and intermolecular amino acids (red) in the vicinity of Leu44 (white). **(c)** A plane from a 3D ^{13}C-edited NOESY spectrum (blue) and 3D ^{12}C-filtered,^{13}C-edited NOESY spectrum (red) highlighting NOEs from the HD2 methyl group of Leu44.

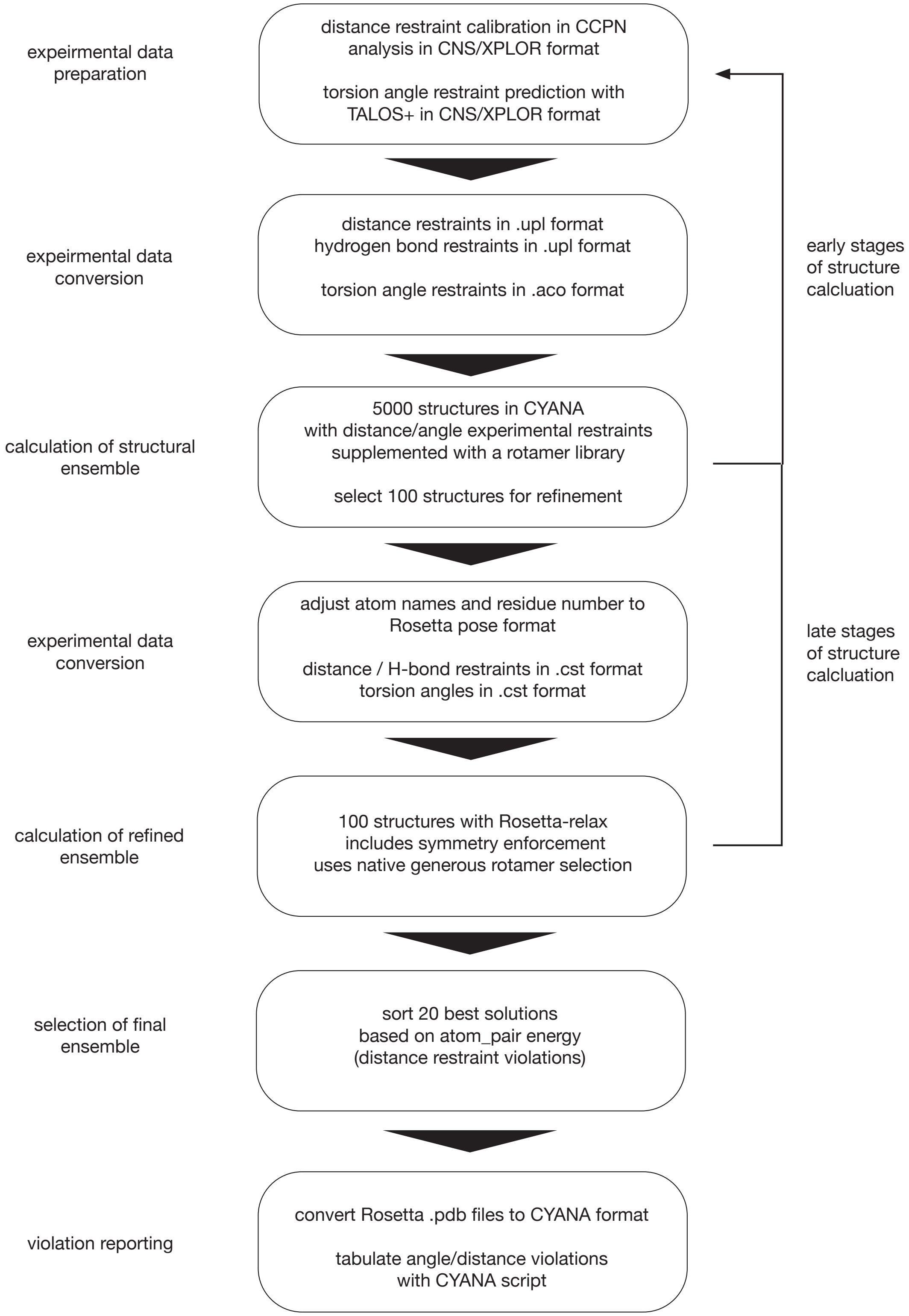

Figure S6 — The CYANA/Rosetta workflow used to determine the solution structure of Orf63. A set of short scripts written in Perl facilitate PDB conversion (renumbering, atom renaming) and restraint conversion between XPLOR, CYANA and Rosetta formats.

a

```
#!/bin/csh

relax.macosclangrelease                    \
-relax:constrain_relax_to_start_coords \
-relax:coord_constrain_sidechains      \
-relax:ramp_constraints false          \
-relax:fast                            \
-s model_{$1}.pdb                      \
-ex1                                   \
-ex2                                   \
-use_input_sc                          \
-flip_HNQ                              \
-fix_his_tautomer 6 27                 \
-no_optH false                         \
-nstruct 1  &
```

b

```
#!/bin/csh

set i = 1
while ($i <= 100)

/Applications/Darwin/rosetta_bin_mac_2021.16.61629_bundle/
main/source/src/apps/public/symmetry/make_symmdef_file.pl -p
model_{$i}_0001.pdb -a A -i B > model_{$i}_0001.symm

@i++
end
```

Figure S7 — Scripts to perform prepare CYANA models for refinement. **(a)** This script prepares one CYANA model for a Rosetta structure calculation by performing a regularization. By default, Rosetta will arbitrarily change the tautomeric state of histidine so a flag must be added to suppress this behaviour. **(b)** This script creates the symmetry file for the dimer calculation.

```
#!/bin/csh
relax.macosclangrelease                                              \
-s ../template/model_{$1}_0001_INPUT.pdb                             \
-symmetry:symmetry_definition ../template/model_{$1}_0001.symm \
-relax:fast                                                          \
-relax:ramp_constraints true                                         \
-relax:jump_move true                                                \
-out:file:scorefile score_$1.sc                                      \
-cst_fa_file ../cst/total.cst                                        \
-cst_weight    0.5                                                   \
-cst_fa_weight 0.5                                                   \
-fix_his_tautomer 6 27                                               \
-ex1                                                                 \
-ex2                                                                 \
-ex2aro                                                              \
-nstruct 1                                                           \
> p{$1}.log &
```

Figure S8 — Rosetta script to perform restraint based refinement of Orf63. This script above processes models from previously regularized CYANA models. This script is wrapped in another shell script enabling 10 refinements to be performed at once on a 10 CPU MacBookPro M1. By default, default will arbitrarily change the tautomeric state of histidine so a flag must be added to suppress this behaviour. Some experimentation is required to select the best weighting for the distance and angle restraints contained in one large .cst file. In practice, this weight is in the range 0.5-2.0.

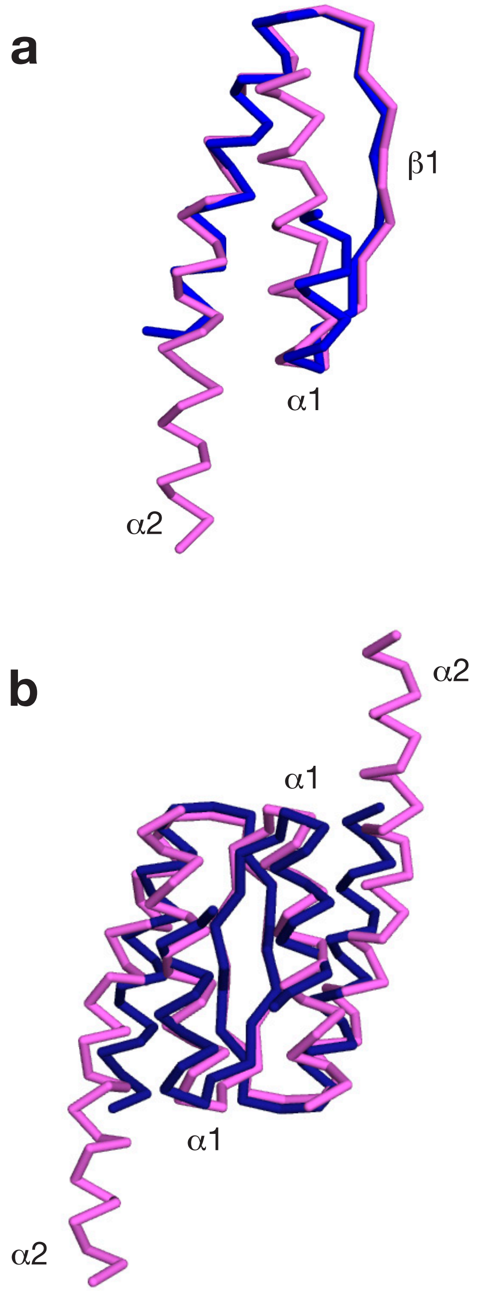

Figure S9 — A comparison of the NMR solution structure (blue) and AlphaFold predicted structure of Orf63 (purple). **(a)** An individual protomer superimposes with a $C\alpha$ RMSD of 1.80 Å. (b) The dimer superimposes with a $C\alpha$ RMSD of 2.95 Å **(b)** AlphaFold predicts longer helix $\alpha 1$ (extended at the N-terminus) and a longer helix $\alpha 2$ (extended at the C-terminus).

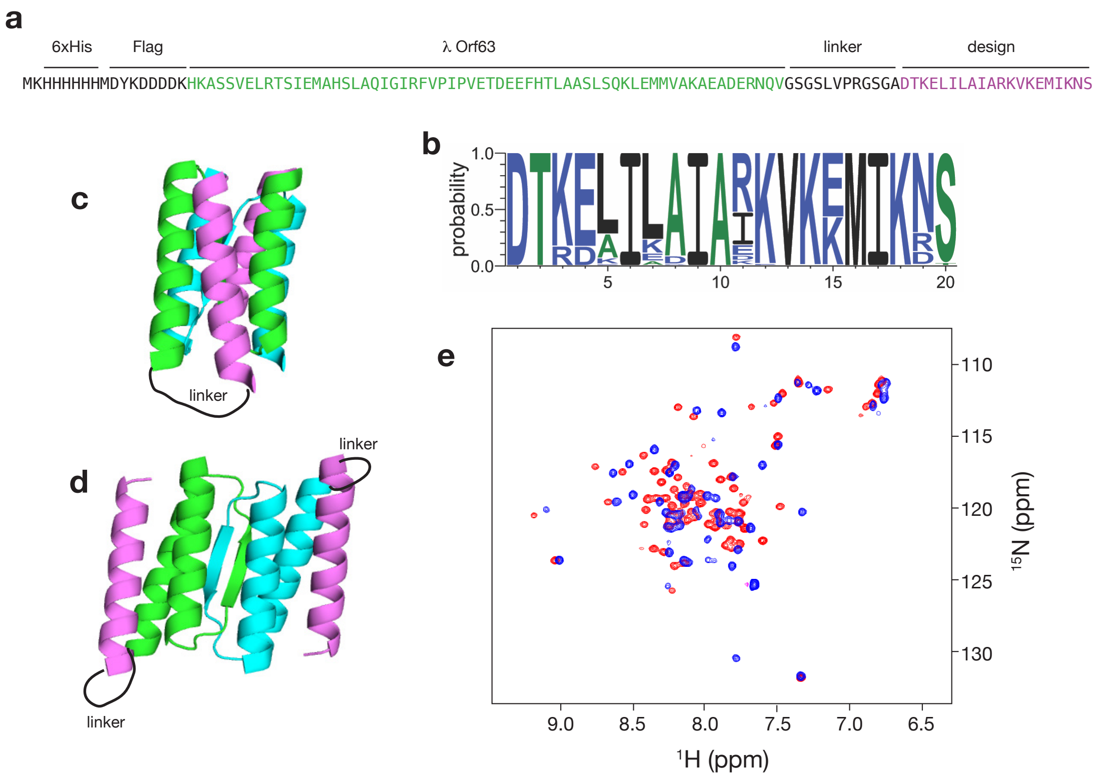

Figure S10 — An NMR investigation of a hybrid Orf63 protein with a designed α-helix appended to the C-terminus. **(a)** Sequence of the hybrid protein. **(b)** Sequence variation throughout the designed helix sampled by Rosetta. **(c,d)** Cartoon representations of the designed helix (purple) against each Orf63 protomer (green/blue). **(e)** A ^{1}H-^{15}N HSQC spectrum of a hybrid Orf63 protein (red) superimposed upon the spectrum of Orf63 (blue).

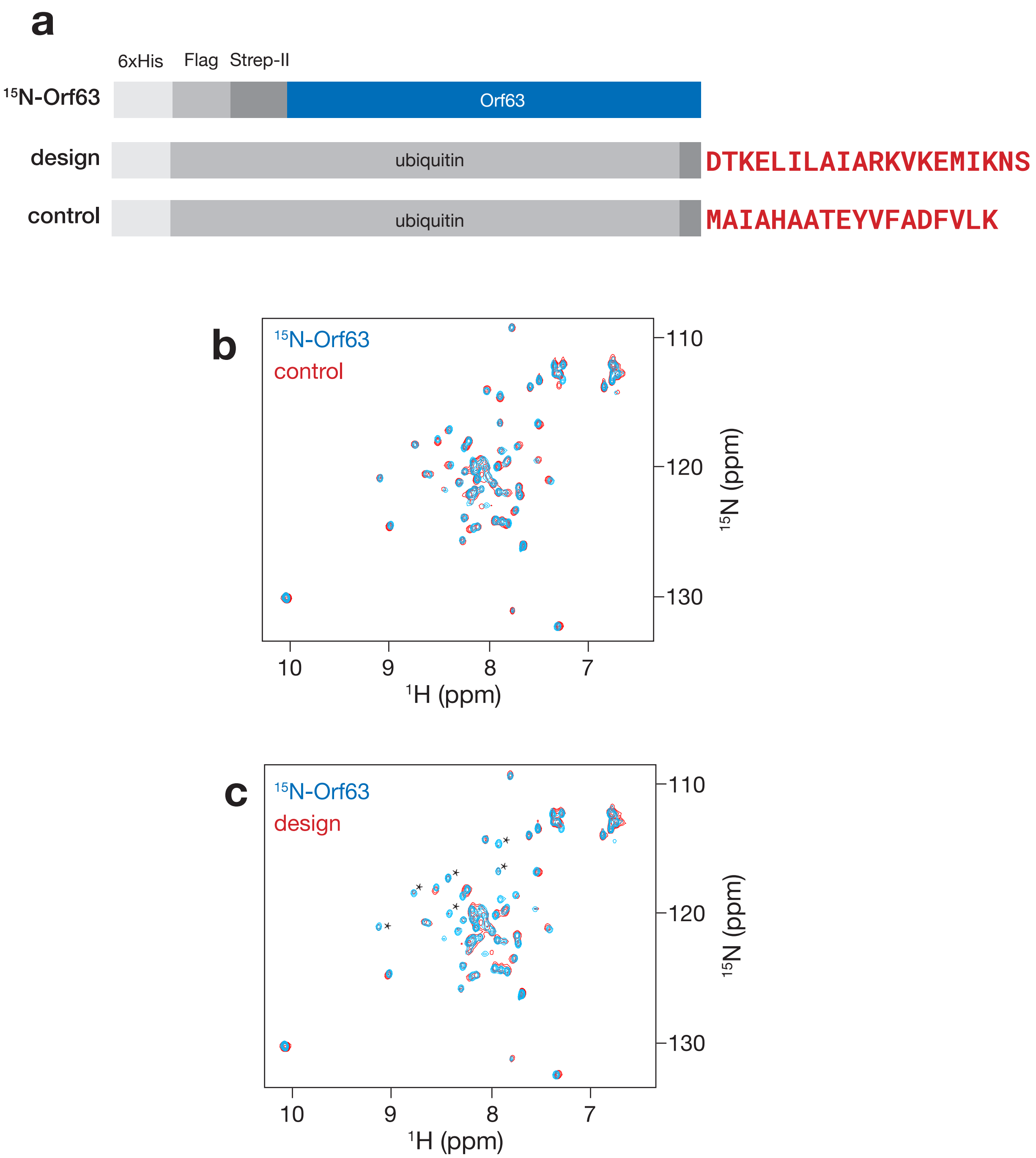

Figure S11 — A designed peptide binds Orf63. **(a)** A mixing experiment was performed with ^{15}N-Orf63 and a Ubiquitin-fusion protein with a C-terminal extension corresponding to a designed peptide, or an unrelated peptide control. The mixing ratio was 2:1 (Ubiquitin fusion to Orf63 dimer) in 5 mM Tris-Cl pH 7.4, 50 mM NaCl. **(b,c)** ^{15}N-HSQC spectra of ^{15}N-Orf63 (blue) and ^{15}N-Orf63 mixed with a Ubiquitin fusion protein. Asterisks denote peaks that were severely line broadened in the mixing experiment.

```
              1         2         3         4         5         6         7
12345678901234567890123456789012345678901234567890123456789012345678901234567 expressed sequence
HHHHHHMDYKDDDDKHKASSVELRTSIEMAHSLAQIGIRFVPIPVETDEEFHTLAASLSQKLEMMVAKAEADERNQV
               23456789012345678901234567890123456789012345678901234567890123 native sequence
                      1         2         3         4         5         6
```

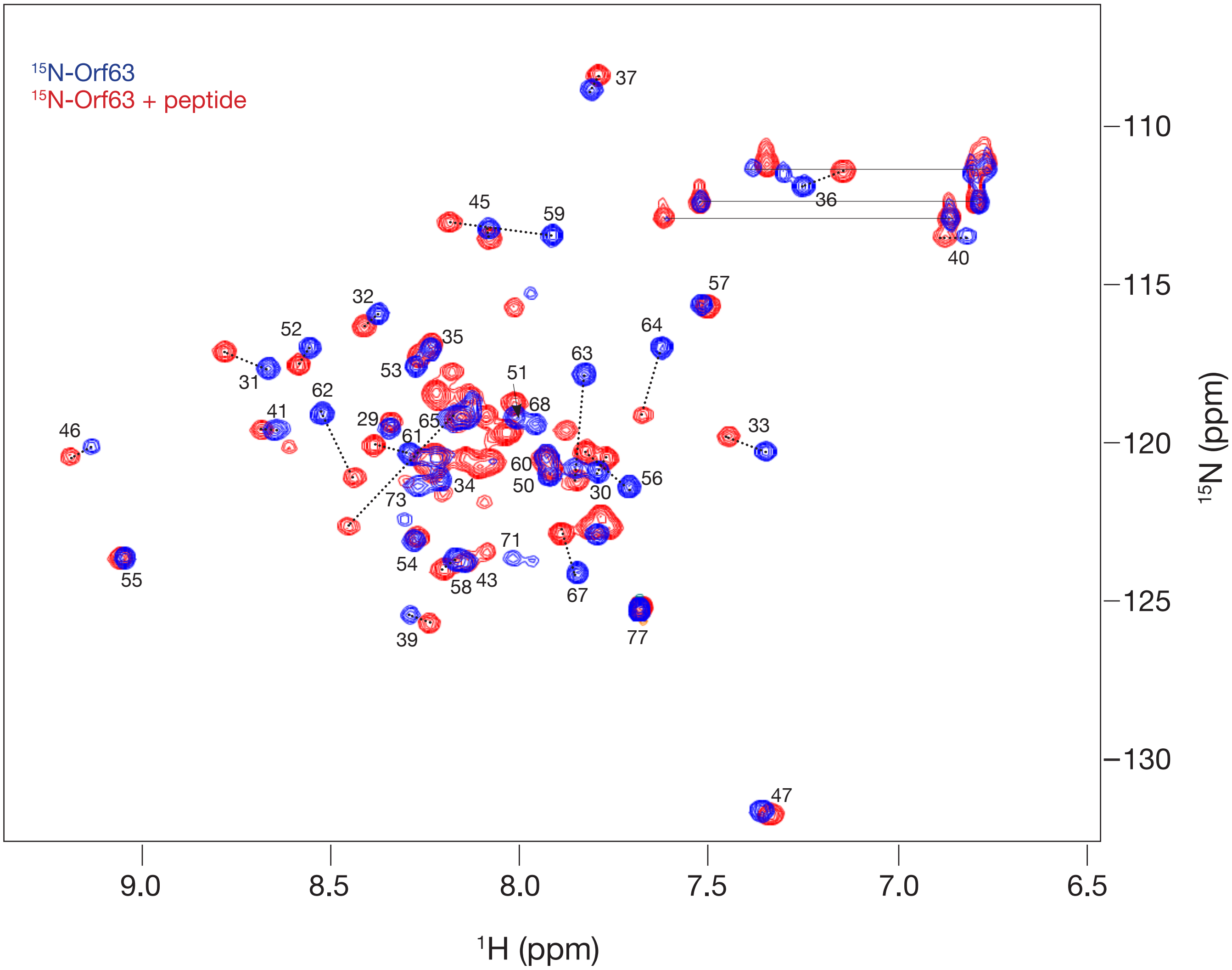

Figure S12 — A designed peptide binds Orf63. ^{15}N-HSQC spectra of ^{15}N-labeled Orf63 (blue) and a 1:1 mixture of peptide to Orf63 protomer (red). Most resonances during the titration exhibited slow exchange behaviour. The peptide bound form of Orf63, especially throughout the two α-helical regions, was confirmed by an HN-HN walk through a 3D ^{15}N-NOESY spectrum. Unassigned resonances N-terminal helix α1 and C-terminal to helix α2 are likely unstructured due to the absence of *i*,*i*+3, *i*,*i*+4, and HN-HN NOEs. The residue numbering follows the PDB file 8DSB and the expressed protein with N-terminal affinity tags.
